# Alterations of lipoprotein subfractions in GH-deficient adults

**DOI:** 10.3389/fendo.2025.1696426

**Published:** 2025-11-05

**Authors:** Balázs Ratku, Hajnalka Lőrincz, Sára Csiha, Lajos Bíró, Annamária Erdei, Eszter Berta, Dóra Ujvárosy, Miklós Bodor, Endre V. Nagy, Zoltán Szabó, Mariann Harangi, Sándor Somodi

**Affiliations:** ^1^ Institute of Health Studies, Faculty of Health Sciences, University of Debrecen, Debrecen, Hungary; ^2^ Department of Emergency Medicine, Faculty of Medicine, University of Debrecen, Debrecen, Hungary; ^3^ Division of Metabolism, Department of Internal Medicine, Faculty of Medicine, University of Debrecen, Debrecen, Hungary; ^4^ Department of Clinical Basics, Faculty of Pharmacy, University of Debrecen, Debrecen, Hungary; ^5^ Division of Endocrinology, Department of Internal Medicine, Faculty of Medicine, University of Debrecen, Debrecen, Hungary

**Keywords:** adult growth hormone deficiency, dyslipidemia, high-density lipoprotein, lipoprotein subfractions, sphingosine 1-phosphate, insulin-like growth factor 1

## Abstract

**Introduction:**

Dyslipidemia is a common complication of adult growth hormone deficiency (AGHD) and considered an important contributor to increased mortality. Previous studies mainly focused on quantitative assessment of lipoproteins, but lipoprotein subfractions and their relationship with insulin-like growth factor 1 (IGF-1) have not been explored.

**Purpose:**

To perform a comprehensive evaluation of lipoprotein subfractions and measuring apolipoprotein L1 (apoL1), sphingosine 1-phosphate (S1P) and apolipoprotein M (apoM) in AGHD.

**Materials and methods:**

11 GH-substituted (GHS) patients, 9 GH-unsubstituted (GHU) patients and 37 controls were included in the study. Lipoprotein subfractions were separated by the Lipoprint system. ApoL1, apoM and S1P were determined by ELISA. In the GHS patients GH-replacement was discontinued for 2 months. Measurements were performed before GH-discontinuation, at the end of the 2-month GH-withdrawal, and 1 month after reinstituting GH-replacement.

**Results:**

Standard lipid parameters, apoM and apoL levels were not different between the groups. GHU patients demonstrated lower apolipoprotein A1 compared to controls (p=0.02) and higher apolipoprotein B100 compared to GHS (p=0.02). GHU and GHS showed higher S1P levels compared to controls (p=0.04 and p=0.01, respectively). Both GHU and GHS patients also presented higher percentage of intermediate-density lipoprotein (IDL) compared to controls (p=0.03 and p=0.01, respectively). Mean LDL size was lower in GHU compared to GHS (p=0.04). Percentage of intermediate HDL was lower in GHU and GHS compared to controls (p<0.001 and p<0.01, respectively). GHU demonstrated higher percentage of small HDL than controls (p<0.001). Overall, log_10_IGF-1 correlated positively with the percentage of large HDL (r=0.27; p=0.04) and intermediate HDL (r=0.38; p<0.01) and negatively with the percentage of small HDL (r=-0.46; p<0.01). Log_10_IGF-1 was the best predictor of small HDL (standardized β=-0.46; p<0.001) in overall subjects. In the GH-withdrawal study, the amount of HDL-6 increased with GH-withdrawal (p=0.03) and the percentage of IDL increased with reinstitution (p=0.05).

**Conclusion:**

Despite no changes in standard lipid parameters, considerable alterations of lipoprotein subfractions were revealed in GH-deficient adults indicating that lipoprotein subfraction analysis may allow for a more precise cardiovascular risk assessment in AGHD. Associations between HDL subfractions and Log_10_IGF-1 demonstrate a novel insight into the role of GH in lipid metabolism.

## 1 Introduction

Adult growth hormone deficiency (AGHD) is characterized by a cluster of cardiovascular risk factors, including visceral adiposity, dyslipidemia, insulin resistance, endothelial dysfunction and low-grade inflammation (1). Based on database studies, as much as 43% of untreated and 32% of treated AGHD patients receive lipid-lowering medications (2), and dyslipidemia is proved to be the most prevalent metabolic complication among GH-unreplaced adults (3). Since literature data suggest that excess mortality in AGHD is mainly due to cardiovascular and cerebrovascular disorders (4, 5), dyslipidemia, with its well-established causal role in these diseases (6), is speculated to be the strongest contributor to the increased mortality (7, 8).

In the past decades, several studies have evaluated the effect of GH deficiency and GH replacement therapy (GHRT) on the lipid profile, although the vast majority of these investigations focused solely on a quantitative analysis of lipoproteins (7). A huge number of observations suggest that assessment of the quality of lipoproteins might be more informative than a simple measurement of high-density lipoprotein cholesterol (HDL-C) and low-density lipoprotein cholesterol (LDL-C) levels (9, 10). Lipoproteins are comprised of different subfractions, which have been demonstrated to have distinct effects on cardiovascular risk (7, 10). This observation has led to a growing interest in subfractionating lipoprotein particles (11). Predominance of small-dense LDL (sdLDL) subfraction has been recognized as an independent cardiovascular risk factor and found to be a feature of subjects at very high risk of cardiovascular diseases (10, 12). Data on sdLDL levels in AGHD patients is very limited. Although reduced GH secretion in obesity was associated with smaller mean LDL size (13), previous studies did not detect changes in LDL size and sdLDL levels in AGHD (14, 15). Alterations in the distribution of HDL subfractions are also commonly found in atherosclerosis-related conditions (9, 16). Current evidence suggests that large HDL subfractions are inversely associated whereas small HDL subfractions are positively associated with the cardiovascular risk (9, 16). Data are inconsistent on total HDL-C levels in AGHD (7), and comprehensive evaluation of HDL subfractions involving comparisons with healthy subjects has not been performed.

Besides HDL-C levels and structure, protein composition of HDL also found an important determinant of HDL-induced atheroprotection (17). Apolipoprotein L1 (apoL1) is a relatively unexplored apolipoprotein, which mainly derives from the liver (17, 18). Literature data suggest that apoL1 is associated with type 2 diabetes mellitus (T2DM) (19), and related to impaired HDL metabolism in insulin resistant states (20). ApoL1 has previously been identified as a GH-responsive marker in healthy athletes receiving daily GH injections (21), but until now it has not been evaluated in GH-deficient adults.

Sphingosine 1-phosphate (S1P) is a lipid mediator with key effects in the cardiovascular system (22, 23). S1P exerts its effects on five S1P receptors (S1P1-5) from which S1P1–3 are mostly expressed in the cardiovascular tissues (23, 24). The role of S1P in cardiovascular physiology is highly complex as it appears to mediate both pro- and antiatherogenic processes (23, 24). About 60% of S1P in plasma are carried on HDL, the remaining is transported by albumin, LDL and very-low-density lipoprotein (VLDL) (25). Apolipoprotein M (apoM) has been recognized as the carrier and a potential modulator of S1P (26). Since HDL is known to have several beneficial properties, including antioxidation, anti-inflammation and vasodilation and S1P also possesses many of these properties, S1P is suggested to contribute to these vasculoprotective functions of HDL (23, 27). Although, disturbed apoM/S1P regulation has been linked to endothel dysfunction and atherosclerosis (28), data on apoM and S1P levels in AGHD is currently lacking.

In the present study we aimed to perform an in-depth evaluation of lipoprotein subfractions as well as apoL1, S1P and apoM levels in GH-unsubstituted (GHU), GH-substituted (GHS) patients and healthy controls. To evaluate possible changes to short-term treatment discontinuation, we also carried out a prospective GH-withdrawal study on a small cohort of GHS patients (n=11). We hypothesized that GHU patients would present severe alterations in lipoprotein subfractions, and given that short-term discontinuation caused deterioration even in the standard lipid parameters (29), we expected marked modifications in lipoprotein subfractions following GH-withdrawal.

## 2 Materials and methods

### 2.1 GH-deficient patients

A total of 20 patients (11 GHS and 9 GHU patients) with well-documented AGHD were recruited from the Endocrinology Outpatient Clinic, Clinical Centre, University of Debrecen, Hungary. Diagnosis of AGHD was based upon a peak GH response to insulin tolerance test less than 3 μg/L during adequate hypoglycemia (30). As for the etiology, five enrolled AGHD patients had craniopharyngioma, four patients had empty sella syndrome, 3 patients had non-functioning pituitary adenoma, while the remaining patients had functioning pituitary adenoma (n=2), astrocytoma (n=2), idiopathic AGHD (n=2), Sheehan’s syndrome (n=1), and ependymoma (n=1). Patients were not eligible for the study if they had active malignant disease, heart failure, kidney failure, liver failure, respiratory insufficiency, were pregnant or breastfeeding.

GHS patients received continuous GH substitution for at least 1 year at the time of enrollment, while GHU patients were unsubstituted for at least 2 years before entering the study. Each AGHD patient, including GHU and GHS subjects suffered multiple pituitary hormone deficiencies and received appropriate thyroid, adrenal and gonadal hormone substitution, as required by their hormonal condition. Age, gender ratio, and the proportion of childhood-onset and adult-onset patients were not significantly different in the GHU and GHS groups. In the GHS group, the mean duration of GH replacement therapy (GHRT) was 18.73 ± 11.76 years, the mean daily GH dose was 0.31 ± 0.15 mg. Clinical characteristics of the enrolled AGHD patients are presented in **Supplementary Table 1**.

### 2.2 Controls

Thirty-seven healthy volunteers (17 males and 20 females) participated in the study as controls (CON). They were carefully selected to match the GHU and GHS groups in terms of age and gender.

### 2.3 Study design

This study employed a combination of a cross-sectional and a prospective design. A detailed description of the study protocol was provided in our previous paper (31) and a flowchart of the study design is shown in **Supplementary Figure 1**. In brief, during the cross-sectional study, all 57 participants (GHS, GHU and CON groups) underwent routine laboratory tests, anthropometric measurements, and determination of lipoprotein subfractions. After these baseline tests (Visit 1), GHS patients (n=11) were enrolled in a prospective GH-withdrawal study, where the same measurements were obtained at two more time points (Visit 2 and 3). GHRT was stopped on the day of Visit 1, and the patients were progressed to a 2-month GH-withdrawal period. All other hormonal replacement therapies were kept unchanged during the withdrawal period. The second visit (Visit 2) was performed after the 2-month withdrawal period. After Visit 2 patients immediately returned to their baseline GH-doses and then called back for a third set of measurements (Visit 3) one month following reinstitution of GHRT.

### 2.4 Ethics

Regional Ethics Committee of the University of Debrecen reviewed and approved the study protocol (registration number: RKIB/IKEB 5576-2020, date of approval: October 27, 2020). Participants were enrolled after oral and written information and provided written informed consent. The study was performed according to the Declaration of Helsinki.

### 2.5 Measurements

#### 2.5.1 Anthropometric measurements

Height was measured in centimeters with a calibrated stadiometer. Body weight was measured during body composition analysis, as described previously (31). Body mass index (BMI) was calculated as weight/height squared (kilograms/meter^2^). Waist circumference (WC) was measured in the standing position midway between the iliac crest and the lower margin of the last palpable rib.

#### 2.5.2 Measurement of routine laboratory parameters

Venous blood samples were collected into Vacutainer tubes after an overnight fast, and sera were prepared immediately. Three aliquots of serum, each approximately 300 μL, were frozen at -80 °C for subsequent measurements. Routine laboratory parameters, including insulin-like growth factor 1 (IGF-1), triglyceride, total cholesterol (TC), LDL-C, HDL-C, apolipoprotein A1 (apoA1), apolipoprotein B100 (apoB100), lipoprotein (a), high sensitivity C-reactive protein (hsCRP), fasting glucose, insulin, hemoglobin A1C (HbA1C), estimated glomerular filtration rate (eGFR) measurements were carried out using a Cobas c600 autoanalyzer (Roche Ltd., Mannheim, Germany) from fresh sera according to standard laboratory protocols at the Department of Laboratory Medicine, Faculty of Medicine, University of Debrecen.

#### 2.5.3 Quantification of LDL subfractions

Lipoprotein subfraction analysis was performed using the Lipoprint system (Quantimetrix Corporation, Redondo Beach, CA, USA) according to the protocol provided by the manufacturer. In brief, 25 µL of the serum was mixed in polyacrylamide gel tubes with 200 µL of lipophilic dye containing a loading gel. Tubes were photopolymerized at room temperature for 30 min and then electrophorized at 3 mA/tubes for 64 min. Each electrophoresis chamber involved a quality control provided by Quantimetrix (Liposure Serum Lipoprotein Control, Quantimetrix Corp., Redondo Beach, CA, USA). For quantification, scanning was done with an ArtixScan M1 digital scanner (Microtek International Inc., CA, USA) and analyzed with the Lipoware Software (Quantimetrix Corp., Redondo Beach, CA, USA). Up to seven LDL subfractions were distributed based on their size between the VLDL and HDL peaks. If present, intermediate density lipoprotein (IDL) particles were represented as Midband C, B and A peaks. The percentage of large LDL (large LDL %) was defined as the summed percentages of LDL1 and LDL2, whereas the percentage of small LDL (small-dense LDL %) was defined as the sum of LDL3–LDL7. Cholesterol concentrations of LDL subfractions were determined by multiplying the relative area under the curve (AUC) of subfractions by the total cholesterol concentration.

#### 2.5.4 Quantification of HDL subfractions

HDL subfractions were distributed using the Lipoprint System (Quantimetrix Corp., Redondo Beach, CA, USA) according to the manufacturer’s protocol. Briefly, 25 µL of the serum was mixed in polyacrylamide gel tubes with 300 µL of lipophilic dye containing a loading gel. From this point, the protocol was identical to that of LDL subfraction test. Ten HDL subfractions were detected based on their size; large (HDL-1 to HDL-3), intermediate (HDL-4 to HDL-7), and small (HDL-8 to HDL-10) HDL subfractions between VLDL + LDL and albumin peaks, respectively. For the determination of the amount of cholesterol (mmol/L) in each subfraction, the cholesterol content of HDL subfractions were calculated by multiplying the AUC of the HDL-C.

#### 2.5.5 Measurement of apoM

Serum apoM levels were measured by a commercially available enzyme linked immunosorbent assay (BioVendor, Czech Republic, Brno, Cat. No. RD191129200R) with 0.07 ng/mL minimum detectable dose. Samples run in 1000x dilution on the plate and values were expressed as μg/mL.

#### 2.5.6 Measurement of apoL1

Concentrations of apoL1 were measured by a commercially available sandwich ELISA (Novus Biologicals LLC a Bio-Techne Brand, Centennial, CO, USA; Cat. No. NBP-27222). The minimum detectable dose of apoL1 was 0.19 ng/mL. Sera were applied in 1000x dilution and values were expressed as μg/mL.

#### 2.5.7 Measurement of S1P

Serum S1P levels were determined using a competitive ELISA (Echelon Bioscience, Salt Lake City, UT, USA; Cat. No. K-1900). The assay is not species specific, and it detects S1P from human or animal serum, plasma, tissue homogenate, and cell lysate. Sera were applied in 10x dilution and values were expressed as μM.

### 2.6 Statistical analysis

All descriptive statistical results are expressed as mean ± SD or median and 25th and 75th percentiles. The normality of data was checked using Kolmogorov–Smirnov and Shapiro-Wilk tests. In the case of nonnormally distributed data logarithmic transformation was used before further statistical analysis. Baseline characteristics of GHU, GHS and CON groups were compared using ordinary one-way analysis of variance (ANOVA) and Tukey’s *post hoc* test. The within group effect of discontinuing GH replacement was analyzed using ANOVA for repeated measurements. Greenhouse-Geisser correction was used for all variables. Eta-squared (η²) values and their corresponding 95% confidence intervals (95% CI) are reported as measures of effect sizes. Pearson’s correlation coefficient was used to explore the linear association between IGF-1 levels and lipoprotein subfractions. To determine significant predictor(s) of small HDL, backward stepwise multiple regression analysis was performed. Statistical analyses were performed using Statistica 13.5.0.17 software (TIBCO Software Inc., Tulsa, OK, USA) and Stata statistical software version 18.0 (Stata Corp., Texas, USA). Statistical significance established at p ≤0.05. Graphs were prepared using GraphPad Prism 9.4.1 (GraphPad Prism Software Inc., San Diego, CA, USA).

## 3 Results

**Figure 1:**
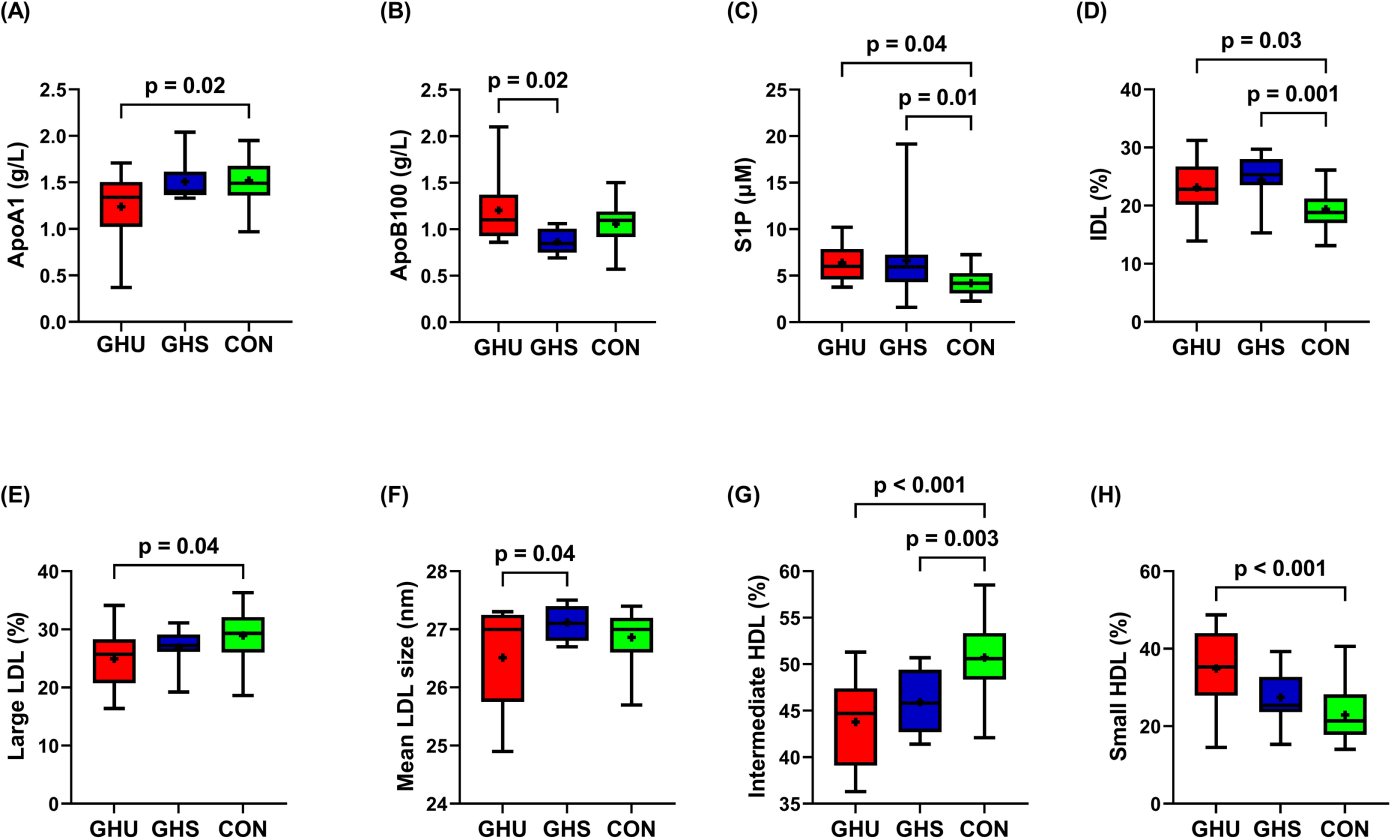
Selected laboratory parameters of the study groups. Box and whisker plots (median, first and third quartiles, minimum and maximum) are used to represent **(A)** apoA1, **(B)** apoB100, **(C)** S1P, **(D)** IDL, **(E)** large LDL, **(F)** Mean LDL size, **(G)** intermediate HDL, **(H)** small HDL in each group. + indicate means. Comparisons were carried out using one-way ANOVA. P values derive from Tukey’s post hoc test. Abbreviations: AGHD, adult growth hormone deficiency; ApoA1, apolipoprotein A1; ApoB100, apolipoprotein B100; CON, healthy control subjects; GHS, GH-substituted GH-deficient subjects; GHU, GH-unsubstituted GH-deficient subjects; HDL, high density lipoprotein; IDL, intermediate-density lipoprotein; LDL, low density lipoprotein; S1P, sphingosine 1-phosphate.

Relevant anthropometric and laboratory parameters of the study participants are presented in **Table 1**. There was no significant difference in terms of age and gender between the three groups. GHU patients had higher BMI compared to both GHS and control subjects, while the BMI was similar between GHS and controls (p>0.99). Waist circumference was higher in GHU than in controls (p=0.02) and approached statistical significance between GHU and GHS (105.8 ± 10.9 cm vs. 89.9 ± 15.2 cm; p=0.06). GHU patients presented lower IGF-1 levels compared to both GHS and controls, while IGF-1 were similar between GHS and CON (p=0.49). hsCRP was higher in GHU than in control subjects (p=0.03) but did not differ between GHU and GHS. Serum glucose levels and HbA1C were not different in the three groups, while insulin levels were lower in the controls compared to both GHU and GHS (p=0.03 and p=0.05, respectively). eGFR was found comparable between the three groups. Similarly, triglyceride, TC, HDL-C and LDL-C levels were not statistically different between the three groups. GHU patients demonstrated lower apoA1 levels compared to controls (p=0.02), while no significant difference was detected between GHU and GHS (**Figure 1A**). ApoB100 level was higher in GHU than in GHS patients, however no difference was found between the patient groups (GHU and GHS) and controls (**Figure 1B**). Levels of apoM, apoL1 and lipoprotein (a) did not show significant difference between the study groups. Compared to controls, GHU and GHS patients showed significantly higher S1P levels (**Figure 1C**).

**Table 1:** Anthropometric and laboratory parameters of the study participants.

Variables	GHU (n = 9)	GHS (n = 11)	CON (n = 37)	P values	η² (95% CI)
Anthropometric parameters					
Male/female (n)	5/4	6/5	17/20	ns	
Age (yrs)	42.2 ± 14.6	43.2 ± 10.2	47.6 ± 10.6	ns	
BMI (kg/m^2^)	32.2 (28.8-39.6)^*^	27.8 (21.6-36.0)^#^	27.1 (22.9-29.3)	0.04^*^ 0.01^#^	0.14 (0.005-0.291)
Waist circumference (cm)	105.8 ± 10.9^#^	89.9 ± 15.2	89.9 **±** 16.1	0.02^#^	0.13 (0-0.279)
Laboratory parameters					
IGF-1 (μg/L)	67.5 (53.6-96.5)^* #^	162.0 (146.0-180.0)	185.2 (153.2-233.4)	<0.01^*^<0.01^#^	0.39 (0.175-0.528)
hsCRP (mg/L)	3.4 (2.7-12.2)^#^	2.1 (1.4-3.5)	1.6 (0.7-3.0)	0.03^#^	0.13 (0-0.275)
Glucose (mmol/L)	4.8 (4.3-5.5)	5.0 (4.7-5.7) (n=10)	5.0 (4.7-5.3)	ns	
HbA1C (%)	5.4 (5.2-5.9)	5.4 (5.2-6.5) (n=10)	5.4 (5.2-5.6)	ns	
Insulin (mU/L)	23.6 (9.9-56.5)^#^	19.9 (9.7-54.1) (n=10)^§^	14.9 (8.1-34.1)	0.03^#^0.05^§^	0.14 (0.002-0.305)
eGFR (mL/1.73 m2)	90.0 (83.5-90.0)	90.0 (78.0-90.0)	83.0 (73.0-90.0)	ns	
Triglyceride (mmol/L)	2.3 (1.5-3.6)	1.7 (1.2-2.6)	1.7 (1.1-2.4)	ns	
Total cholesterol (mmol/L)	5.1 ± 1.4	5.3 ± 0.7	5.8 ± 1.0	ns	
HDL-C (mmol/L)	1.1 ± 0.3	1.2 ± 0.3	1.4 ± 0.4	ns	
LDL-C (mmol/L)	3.2 ± 0.9	3.0 ± 0.6	3.4 ± 0.7	ns	
Apolipoprotein A1 (g/L)	1.24 ± 0.4^#^	1.51 ± 0.2	1.52 ± 0.2	0.02^#^	0.13 (0-0.289)
Apolipoprotein B100 (g/L)	1.20 ± 0.4^*^	0.86 ± 0.3	1.10 ± 0.2	0.02^*^	0.12 (0-0.268)
Apolipoprotein L1 (μg/mL)	12.9 ± 11.8	7.3 ± 3.6	8.1 ± 4.9	ns	
Apolipoprotein M (μg/mL)	2.7 ± 0.8	2.8 ± 0.4	2.8 ± 0.4	ns	
Sphingosine 1-phosphate (μM)	6.4 ± 2.1^#^	6.6 ± 4.5^§^	4.2 ± 1.2	0.04^#^0.01^§^	0.19 (0.027-0.35)
Lipoprotein (a) (mg/L)	56.5 (30.0-324.0)	98.0 (35.0-349.5)	92.0 (46.0-429.0)	ns	

Data are expressed as mean ± SD or median ± interquartile range in case of nonnormally distributed data and analyzed using one-way ANOVA. P values derive from Tukey’s *post hoc* test and are presented if the overall ANOVA has a p value of less than or equal to 0.05. Difference of male/female ratio between groups was analyzed using Chi-square test and Fisher’s exact test. Our T1DM patient in the GHS group has been excluded from the analysis of the parameters of glucose metabolism. *Indicates statistically significant difference between GHU and GHS. ^#^Indicates statistically significant difference between GHU and CON. §Indicates statistically significant difference between GHS and CON. BMI, body mass index; CON, healthy control subjects; eGFR, glomerular filtration rate; GHS, GH-substituted GH-deficient patients; GHU, GH-unsubstituted GH-deficient patients; HbA1C, Hemoglobin A1C; HDL-C, high density lipoprotein cholesterol; HOMA-IR, homeostatic model assessment for insulin resistance; hsCRP, high sensitivity C-reactive protein; IGF-1, insulin-like growth factor 1; LDL-C, low density lipoprotein cholesterol; ns, not significant.

**Figure 2:**
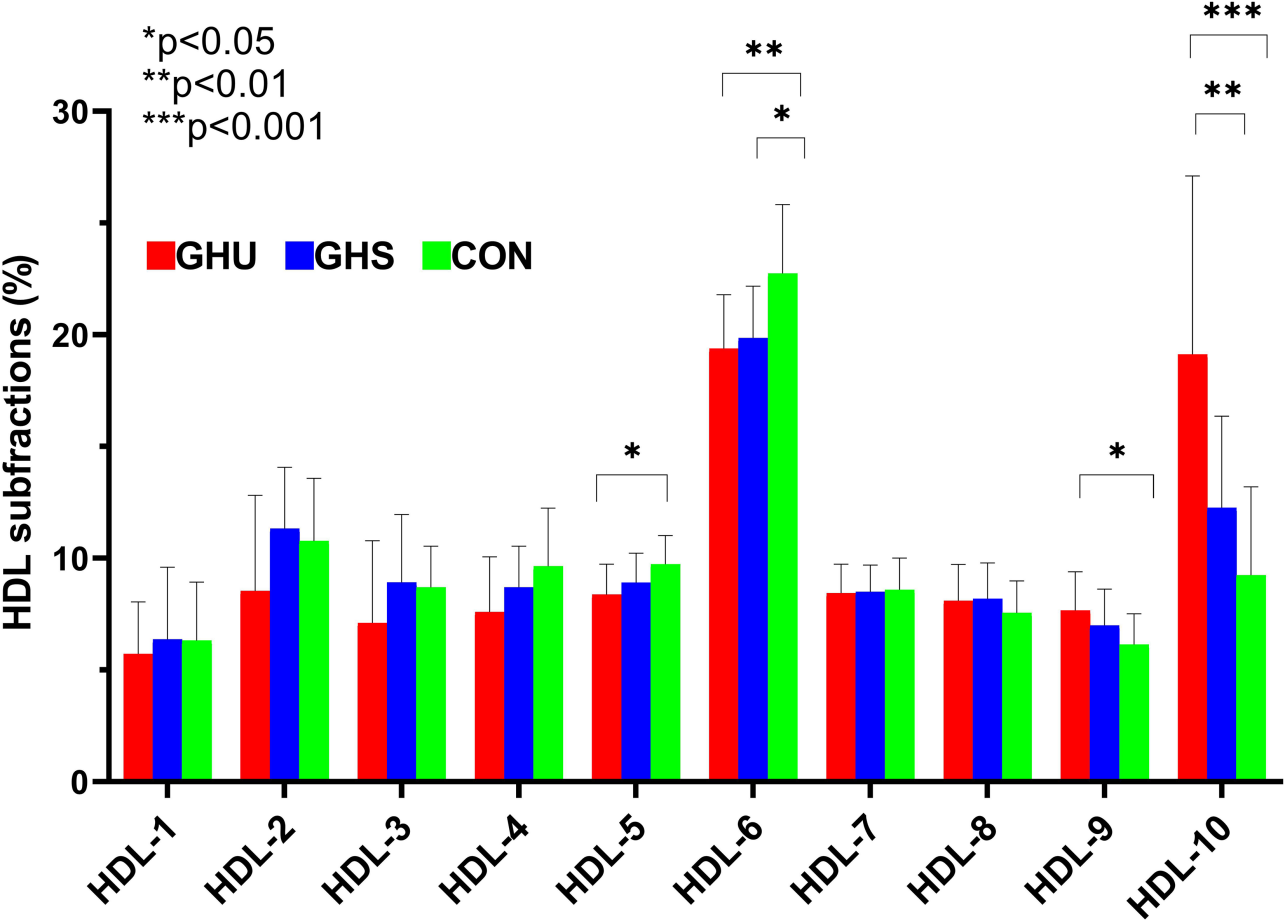
Percentage of individual high-density lipoprotein (HDL) subfractions in GHU (red bars; n=9), GHS (blue bars; n=11) and CON subjects (green bars; n=37). Data are presented as mean ± standard deviation. Differences between the study groups were analyzed using one-way ANOVA. * indicates p < 0.05; ** indicates p < 0.01; *** indicates p< 0.001. CON, healthy control subjects; GHS, GH-substituted GH-deficient subjects; GHU, GH-unsubstituted GH-deficient subjects.

Percentage and absolute amount of lipoprotein subfractions in the study groups are summarized in **Table 2**. Absolute amount and proportion of VLDL did not differ significantly between the three groups, although, a tendency to increased amount of VLDL was observed in GHU compared to GHS (p=0.06). Compared to control subjects, both GHU and GHS demonstrated higher percentage of IDL (**Figure 1D**). In relation to controls, percentage of large LDL was lower in GHU subjects, but did not differ between GHU and GHS (**Figure 1E**). There was a trend towards a higher amount of sdLDL in GHU compared to GHS (p=0.06), but statistically significant difference was not observed between the three groups. Mean LDL size appeared lower in GHU compared to GHS (p=0.04) but did not differ between the patient groups and CON (**Figure 1F**). Percentage of intermediate HDL was markedly higher in controls compared to both GHU (p<0.001) and GHS (p<0.01) (**Figure 1G**), while the absolute amount of intermediate HDL differed only between GHU and CON (p<0.01). GHU patients also showed higher percentage of small HDL than CON subjects (**Figure 1H**). As shown in **Figure 2**, there was a shift towards a greater proportion of smaller and denser HDL particles in GHU subjects. Individual HDL subfractions corresponding to large HDL (HDL-1-3) did not differ between the three groups (**Figure 2**). Among subfractions corresponding to intermediate HDL (HDL-4-7), the percentage of HDL-5 was found lower in GHU compared to controls (p=0.02), while the percentage of HDL-6 was found lower in both GHU and GHS compared to controls (p=0.007 and p=0.01, respectively) (**Figure 2**). Among small HDL subfractions (HDL-8-10), the percentage of HDL-9 was higher in GHU compared to controls (p=0.02), while the percentage of HDL-10 was markedly increased in GHU compared to both GHS (p=0.006) and healthy controls (p<0.0001) (**Figure 2**).

**Table 2:** Percentage and absolute amount of lipoprotein subfractions in the study groups.

Variables	GHU (n = 9)	GHS (n = 11)	CON (n = 37)	P values	η² (95% CI)
VLDL (%)	23.21 ± 6.93	19.24 ± 3.78	20.00 ± 3.64	ns	
IDL (%)	23.13 ± 5.09^#^	24.37 ± 4.36^§^	19.38 ± 3.39	0.03^#^ <0.01^§^	0.25 (0.062-0.401)
Large LDL (%)	24.94 ± 5.34^#^	26.89 ± 3.17	28.94 ± 4.21	0.04^#^	0.09 (0-0.23)
Small-dense LDL (%)	1.9 (0.0-13.3)	1.2 (0.0-2.1)	2.0 (1.1-3.7)	ns	
Mean LDL size (nm)	27.0 (25.8-27.3)*	27.1 (26.8-27.4)	27.0 (26.6-27.2)	0.04*	0.08 (0-0.221)
Large HDL (%)	21.34 ± 8.70	26.62 ± 7.50	26.35 ± 7.28	ns	
Intermediate HDL (%)	43.79 ± 4.86^#^	45.94 ± 3.37^§^	50.71 ± 3.90	<0.001^#^ <0.01^§^	0.33 (0.127-0.479)
Small HDL (%)	34.88 ± 10.93^#^	27.45 ± 7.25	22.94 ± 6.53	<0.001^#^	0.24 (0.06-0.398)
VLDL (mmol/L)	1.26 ± 0.48	0.96 ± 0.16	1.11 ± 0.26	ns	
IDL (mmol/L)	1.27 ± 0.44	1.24 ± 0.32	1.09 ± 0.30	ns	
Large LDL (mmol/L)	1.35 ± 0.40	1.35 ± 0.26	1.16 ± 0.40	ns	
Small-dense LDL (mmol/L)	0.10 (0.00-0.58)	0.05 (0.00-0.10)	0.11 (0.06-0.24)	ns	
Large HDL (mmol/L)	0.25 ± 0.18	0.36 ± 0.17	0.38 ± 0.18	ns	
Intermediate HDL (mmol/L)	0.49 ± 0.17^#^	0.60 ± 0.12	0.70 ± 0.16	<0.01	0.18 (0.024-0.34)
Small HDL (mmol/L)	0.37 ± 0.12	0.35 ± 0.07	0.31 ± 0.07	ns	

Data are presented as mean ± SD or median (interquartile range) in case of nonnormally distributed data and analyzed using one-way ANOVA. P-values derive from Tukey’s *post hoc* test and presented if the overall ANOVA has a p value of less than or equal to 0.05. * indicates statistically significant difference between GHU and GHS. # indicates statistically significant difference between GHU and CON. § indicates statistically significant difference between GHS and CON. CON, healthy control subjects; GHS, GH-substituted AGHD patients; GHU, GH-unsubstituted AGHD patients; HDL, high-density lipoprotein; IDL, intermediate-density lipoprotein; LDL, low-density lipoprotein; ns, not significant; VLDL, very-low density lipoprotein.

**Figure 3:**
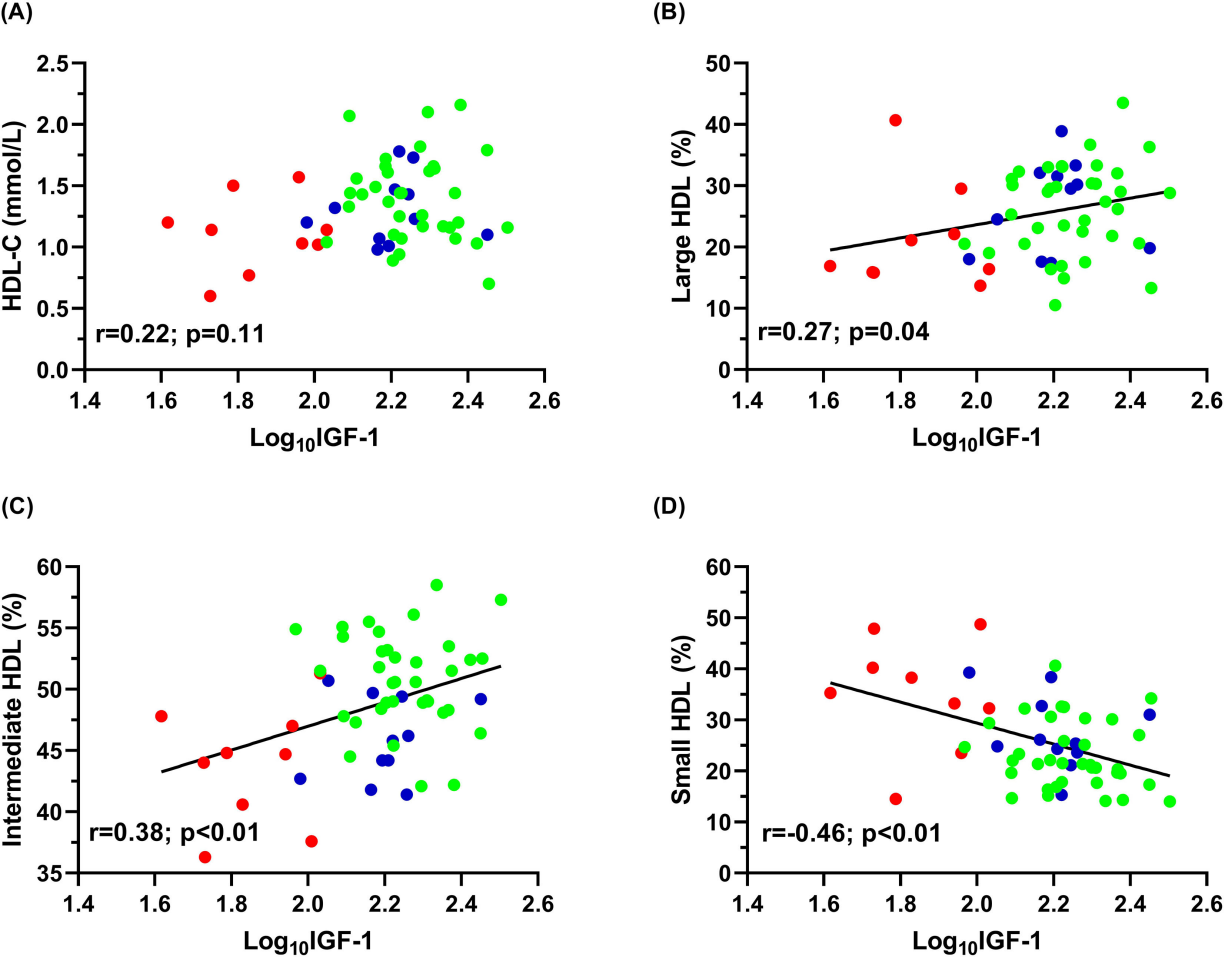
Data of Pearson’s correlations of log_10_IGF-1 with **(A)** HDL-C; **(B)** large HDL; **(C)** intermediate HDL and **(D)** small HDL in GHU (red; n=9), GHS (blue; n=11) and control subjects (green; n=37). GHS, GH-substituted GH-deficient subjects; GHU, GH-unsubstituted GH-deficient subjects; HDL-C, high-density lipoprotein-cholesterol; IGF-1, insulin-like growth factor 1.

There were no correlations between log_10_IGF-1 and LDL-C or LDL subfractions. Despite no significant correlations were observed between HDL-C and log_10_IGF-1 (**Figure 3A**), log_10_IGF-1 showed significant positive correlation with the percentage of large and intermediate HDL (**Figures 3B, C**) and strong negative correlation with the percentage of small HDL in overall subjects (**Figure 3D**).

Backward stepwise multiple regression analysis was performed to determine the predictor of small HDL, which is a well-established marker of HDL functionality. The model included BMI, age, gender, waist circumference, log_10_IGF-1, log_10_CRP, log_10_inzulin and S1P. In the overall subjects, the significant predictor of small HDL was Log_10_IGF-1 (standardized β=-0.46; p<0.001).

**Figure 4:**
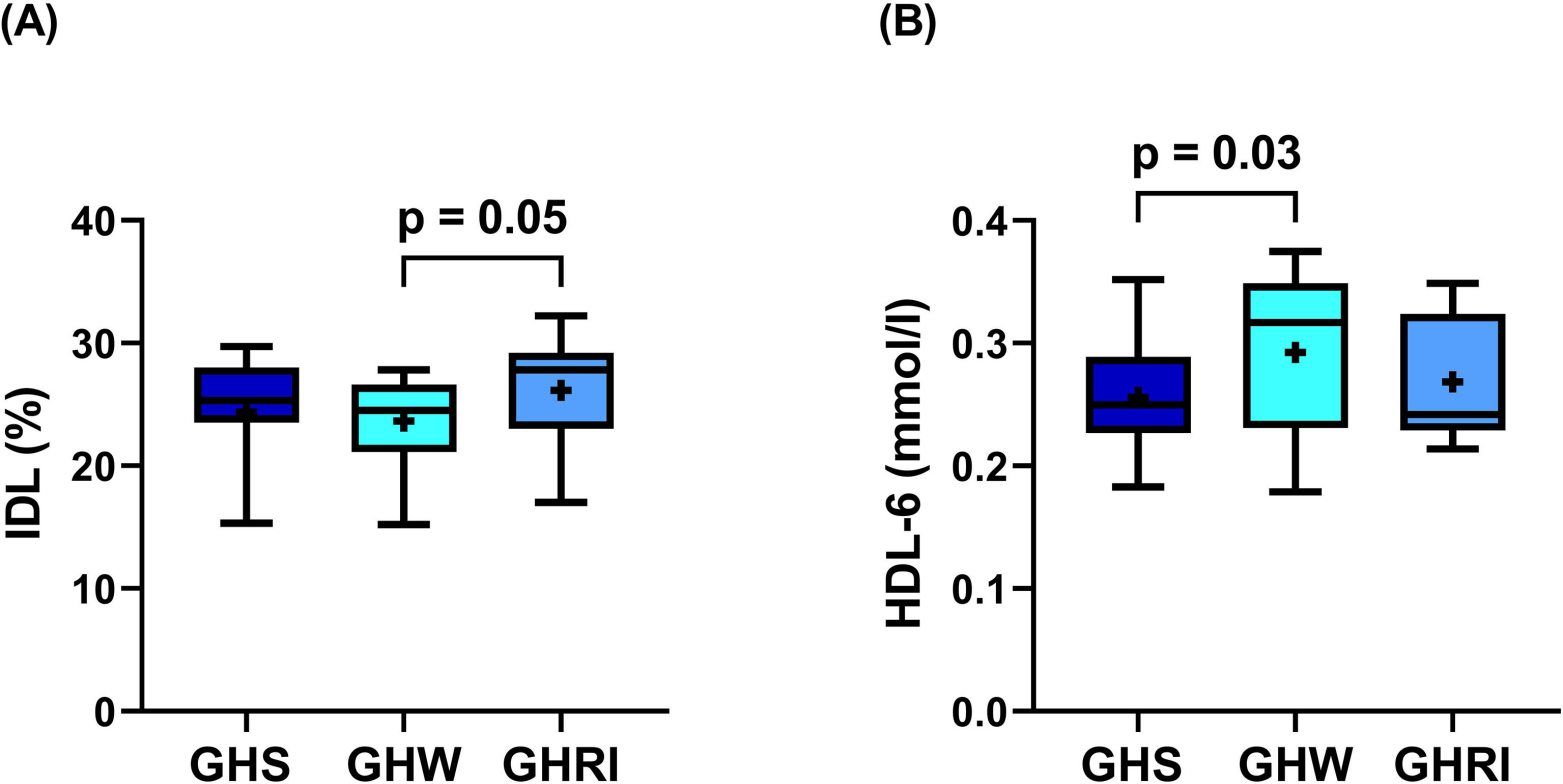
Changes during GH-withdrawal and reinstitution. Box and whisker plots (median, first and third quartiles, minimum and maximum) are used to represent changes of the **(A)** IDL and **(B)** HDL-6 during continuous GH-replacement (GHS), after 2-month of GH-withdrawal (GHW), and following one month of GH-reinstitution (GHRI). + indicate means. Changes were analyzed using ANOVA for repeated measures. P values derive from Tukey’s post hoc test. Abbreviations: AGHD, adult growth hormone deficiency; GH, growth hormone; GHRI, growth hormone reinstitution; GHS, GH-substituted GH-deficient patients; GHW, growth hormone withdrawal; HDL, high-density lipoprotein; IDL, intermediate-density lipoprotein.

As expected, IGF-1 levels decreased after 2-month GH-withdrawal (from 162.0 (146.0-180.0) μg/L to 95.1 (67.4-141.7) μg/L, p<0.01) and then increased significantly after 1 month reinstitution (164.3 (134.2-222.7) μg/L, p<0.01; data not shown). Standard lipid parameters and lipoprotein subfractions were mainly unaffected by withdrawal and reinstitution (**Table 3**) except for the percentage of IDL, which presented a decline after GH-withdrawal and increased with reinstitution (**Figure 4A**), and the absolute amount of HDL-6 subfraction, which showed significant increase after GH-withdrawal (**Figure 4B**).

**Table 3:** Results of 2-month GH-withdrawal and reinstitution on the lipid profile, lipoprotein subfractions and sphingosine 1-phosphate levels.

Variables	GHS (n=11)	GHW (n=11)	GHRI (n=11)	P values
Triglyceride (mmol/L)	1.7 (1.2- 2.6)	1.6 (1.1-2.2)	1.6 (1.2-1.8)	not significant
Total cholesterol (mmol/L)	5.3 ± 0.7	4.9 ± 0.9	5.3 ± 0.8	not significant
HDL-C (mmol/L)	1.2 ± 0.3	1.5 ± 0.3	1.4 ± 0.2	not significant
LDL-C (mmol/L)	3.0 ± 0.6	3.1 ± 0.9	3.1 ± 0.6	not significant
Apolipoprotein A (g/L)	1.51 ± 0.2	1.50 ± 0.2	1.48 ± 0.2	not significant
Apolipoprotein B100 (g/l)	0.86 ± 0.3	1.0 ± 0.2	0.96 ± 0.14	not significant
Apolipoprotein L (μg/mL)	7.3 ± 3.6	12.3 ± 10.7	8.9 ± 3.9	not significant
Apolipoprotein M (μg/mL)	2.8 ± 0.4	2.8 ± 0.4	2.8 ± 0.3	not significant
Sphingosine 1-phosphate (μM)	6.6 ± 4.5	7.5 ± 4.8	7.0 ± 7.1	not significant
VLDL (mmol/l)	0.96 ± 0.16	0.91 ± 0.17	0.95 ± 0.17	not significant
IDL (mmol/l)	1.24 ± 0.32	1.23 ± 0.36	1.35 ± 0.39	not significant
Large LDL (mmol/l)	1.35 ± 0.26	1.41 ± 0.32	1.32 ± 0.23	not significant
Small-dense LDL (mmol/l)	0.05 (0.00-0.10)	0.07 (0.00-0.09)	0.04 (0.00-0.09)	not significant
Large HDL (mmol/l)	0.36 ± 0.17	0.34 ± 0.13	0.31 ± 0.12	not significant
Intermediate HDL (mmol/l)	0.60 ± 0.12	0.68 ± 0.18	0.60 ± 0.12	not significant
Small HDL (mmol/l)	0.35 ± 0.07	0.39 ± 0.07	0.38 ± 0.06	not significant

Data are presented as mean ± SD or median (interquartile range) in case of nonnormally distributed data and analyzed using repeated measures ANOVA. P-values derive from Tukey’s *post hoc* test and presented if the overall ANOVA has a p value of less than or equal to 0.05. GH, growth hormone; GHRI, growth hormone reinstitution; GHS, GH-substituted GH-deficient patients; GHW, growth hormone withdrawal; HDL-C, high-density lipoprotein cholesterol; IDL, intermediate density lipoprotein; LDL-C, low density-lipoprotein cholesterol; VLDL, very-low-density lipoprotein.

## 4 Discussion

Increased LDL and decreased HDL levels are among the main risk factors for cardiovascular disease (32). In fact, the effect of LDL-C and HDL-C on cardiovascular health is not only influenced by their circulating levels but also dictated by the quality of the lipoprotein particles (10, 33). LDL consists of several subclasses that differ in density, size, metabolic function as well as atherogenicity (14). Recent studies have shown that quantification of the LDL subfractions could allow for a more precise evaluation of the cardiovascular risk and provide a proper starting point for therapeutical interventions (34). Increased levels of sdLDL have been shown to be a feature of subjects with highly adverse cardiovascular risk profile, such as patients with coronary artery disease (CAD) and T2DM (12). The possible mechanisms behind the enhanced atherogenicity of sdLDL involve their ability to easily penetrate the arterial wall, heightened oxidative susceptibility and reduced affinity to LDL-receptors resulting in prolonged half-life in the circulation (12). Since antioxidant capacity decreases and oxidative susceptibility increases significantly with decreasing LDL size, evaluating LDL size itself is also considered valuable in the cardiovascular risk assessment (12, 35). This is corroborated by several studies where LDL size was found to be an independent predictor of cardiovascular events as well as progression of CAD (10). Currently, available data on LDL subfractions in patients with AGHD are very limited. In a small study (n=14) using gradient gel electrophoresis, GH-unsubstituted AGHD patients demonstrated a shift towards more dense LDL particles, but the LDL size was unchanged compared to controls (14). In another study employing the same separation method as our research group, no difference was found in the LDL size and sdLDL levels between GH-unreplaced AGHD patients and controls (15). In the present study, we detected smaller mean LDL size in GHU patients compared to their GH-substituted counterparts. sdLDL also appeared higher in GHU subjects compared to GHS patients, but the difference did not reach statistical significance. The proportion of large LDL was found lower in unsubstituted AGHD patients compared to healthy controls. But due to the controversial results from previous studies (34, 36, 37), the clinical significance of this finding is currently unclear.

Subfraction analysis revealed higher proportion of IDL in both patient groups compared to controls, and a tendency to a higher amount of VLDL in GHU patients compared to controls. Importantly, due to their pleiotropic proatherogenic actions, these triglyceride rich lipoproteins (TRLs) are causally related to the development of myocardial infarction and ischemic stroke (38). It has been demonstrated that TRL remnants can cross the endothelium, accumulate in the subendothelial space and can induce inflammation and foam cell formation more potently than LDL (38). In a case control study (n=8), Twickler and colleagues reported elevated postprandial remnant-like particle cholesterol concentrations in AGHD and demonstrated improvement with GH replacement (39). In our study, compared to GHS subjects, GHU patients demonstrated higher levels of apoB100, which is the predominant structural protein within TRLs and their remnants (40). As each TRL particle contains a single apoB100 molecule, higher apoB100 concentration, found in GHU patients, reliably reflects higher levels of atherogenic lipoproteins in the circulation (41).

The inverse relationship between plasma HDL-C levels and the risk of cardiovascular diseases has been reported extensively (9). However, it is now widely accepted that HDL function can also be impaired in certain conditions, indicating that evaluating various measures of HDL functionality (e.g. protein composition, particle size, subclass distribution) might be more important than measuring HDL-C levels (9, 42). To the best of our knowledge, there has been only one study evaluating HDL subfractions in AGHD patients (43), but comparisons between patients and healthy controls have not been reported so far. In the present study, large HDL subfractions, which has been considered cardioprotective (44), and found to be inversely associated with cardiovascular risk (45), did not differ significantly between the three study groups. Conversely, in GHU patients we found higher proportion of small HDL subfractions, which have been detected in many other pathological conditions, including morbid obesity (46), hypertension (47), T2DM (48) and CAD (49). Moreover, in a large-scale prospective study, higher baseline small HDL levels were associated with higher risk of future diabetes (50). Interestingly, in a study of 382 patients who underwent coronary angiography due to angina-like chest pain, small HDL was associated not only with the presence of CAD but also with the severity of the disease (51). Even though the exact mechanism by which small HDL particles contribute to atherosclerosis is unclear, it is noteworthy that in diabetic patients small HDL particles were found primary carriers of ceramides, which can facilitate inflammation and insulin resistance through activating the Nuclear factor-kappa B transcription factor (42, 52).

Proportion of intermediate HDL (HDL-4-7) were lower in the two patient groups compared to controls. As for the proportion of individual HDL subfractions, HDL-5 was lower in GHU patients than in controls, while HDL-6 was found lower in both patient groups compared to controls. Based on previous studies, these alterations in the proportion of intermediate HDL subfractions also indicate unfavorable cardiovascular risk in GH-deficient patients. In a study including patients with newly diagnosed and advanced T2DM, Femlak and colleagues reported worsening cardiac function along with lower intermediate HDL and HDL-6 subfraction in patients with advanced T2DM (53). Likewise, intermediate HDL inversely correlated with the presence and number of affected coronary arteries in patients with verified coronary atherosclerosis (54). Furthermore, compared to healthy subjects, dyslipidemic children and adolescents demonstrated lower HDL-6 and HDL-7 levels as well as lower levels of the sum of intermediate HDL subfractions (55).

Inverse association between IGF-1 and LDL-C levels has long been demonstrated in AGHD patients (56), however, the relationship of IGF-1 with lipoprotein subfractions is not fully elucidated. In our study, no significant associations found between routine lipid parameters and log_10_IGF-1. However, as one of our most remarkable findings, log_10_IGF-1 positively correlated with the proportion of large and intermediate HDL and negatively correlated with the proportion of small HDL, suggesting that physiological IGF-1 levels are associated with a more favorable distribution of HDL subfractions. Previous studies found no consistent effect of AGHD and GHRT on HDL levels suggesting that GH does not affect HDL metabolism (7). Conversely, our findings, based on a comprehensive analysis of lipoprotein subfractions, indicate that GH/IGF-1 axis might influence the structure of HDL particles, even if no changes of total HDL-C levels are detected.

It is important to note that similar abnormalities in HDL and LDL subfractions have also been reported in metabolically healthy (57) and morbidly obese subjects (46). Therefore, higher BMI and waist circumference of our GHU patients apparently limit our ability to determine the extent to which obesity contributes to these alterations. Considering the observed associations between log_10_IGF-1 and HDL-subfractions, we evaluated the predictors of small HDL, which is a well-documented marker of HDL functionality. Supporting the potential role of GH/IGF-1 axis in lipid metabolism, log_10_IGF-1 has been proved the best predictor of small HDL. Furthermore, the non-obese GHS patients also presented some of these changes (decreased intermediate HDL, decreased HDL-6 compared to controls) indicating that these alterations are not fully attributable to obesity.

To the best of our knowledge, this is the first study to evaluate S1P, apoM and apoL1 levels in GH-deficient adults. Despite apoL1 was found to be a GH-responsive biomarker in healthy athletes receiving growth hormone therapy (21), we did not find significant difference regarding apoL1 levels between the three study groups. ApoM levels were also found similar in the two patient groups and controls. The possible reason of unaltered apoM and apoL1 levels in the three groups may lie in their distribution across HDL fractions. Based on previous studies, both apolipoproteins bind predominantly to large HDL subfractions (20, 58), which were also unchanged between the three study groups.

On the contrary, serum S1P levels were higher in both GHU and GHS patients compared to controls. The role of S1P in the development of atherosclerosis has been discussed thoroughly in many high-quality review papers (23, 24, 59). In general, S1P has antiatherogen effects, but depending on whether it is bound to apoM or albumin, it can also exert proatherogenic effects (23, 24). Studies investigating S1P levels have reported inconsistent results. In particular, studies on patients with myocardial infarction demonstrated both lower (60) and unchanged plasma S1P levels (61) compared to healthy controls. On the contrary, S1P was positively associated with the severity of coronary sclerosis and found to be a better predictor of obstructive CAD than traditional risk factors (62). Similarly, transient myocardial ischemia caused by percutaneous coronary intervention resulted a dramatic increase of S1P levels suggesting that S1P might be an early marker of ischemia (63). Endothelial dysfunction has long been reported as a significant contributor to premature atherosclerosis in patients with AGHD (1). S1P has been demonstrated to promote endothelial barrier function through spreading of endothelial cells, stabilizing endothelial cell-cell junctions and stimulating endothelial nitric oxide synthase-derived nitric oxide production (23). In principle, this would indicate that higher plasma S1P levels detected in AGHD patients are beneficial to endothelial function, although the effect of S1P on endothelial barrier is demonstrated to be dependent on the plasma concentration, and increased S1P concentrations (e.g. > 5 μM) caused endothelial disruption (24). Moreover, S1P bound to albumin was demonstrated to induce plasminogen activator inhibitor 1 expression resulting in higher risk of thrombotic conditions (23). In a recent preclinical study on cholesterol-fed apolipoprotein E-deficient mice, long-term S1P elevation produced accelerated atherosclerosis and decreased plaque stability resulting in plaque rupture and atherothrombosis (64). Based on their results, chronically increased S1P may cause defective cholesterol efflux from macrophages and dramatically downregulates the key ATP-binding cassette transporters (ABCA1 and ABCG1) (64). Given the complex pro- and antiatherogenic properties of S1P, further studies ideally involving measurement of apoM-bound and albumin-bound S1P are needed to clarify the role of S1P in AGHD patients.

Considering the marked lipoprotein subfraction alterations found in our GHU patients, in the prospective withdrawal study we expected increasing TRLs and unfavorable changes in HDL subfractions. While standard lipid parameters and lipoprotein subfractions were mostly unaffected by 2-month GH-withdrawal, percentage of IDL and the most predominant intermediate HDL subfraction, HDL-6 changed favorably. These paradoxical changes are unanticipated but not entirely unique in withdrawal studies. In a similar withdrawal study, Maraninchi and colleagues also found decreased plasma TRL concentrations after one month of GH-withdrawal (65). In another study, 4-month withdrawal resulted significant decrease in triglyceride levels (29). Unfortunately, our study cannot identify the exact mechanism behind these seemingly favorable changes during GH-withdrawal. However, improved insulin sensitivity, which has been demonstrated in GH-withdrawal studies (29, 31, 65), could be a reasonable explanation for these findings. It is well-established that GH is a counterregulatory hormone that antagonizes the peripheral and hepatic effects of insulin (66). Similar effects have also been described with administered GH; therefore, it is not surprising that decreased insulin sensitivity related to GHRT has been extensively reported in the literature (67). Hyperinsulinemia and insulin resistant conditions are associated with an overproduction of TRLs (68). Consequently, growth hormone withdrawal and the parallel improvement in insulin sensitivity could result in reduced TRL levels.

No known studies evaluated HDL subfractions during GH-withdrawal. As previous studies found lower HDL-6 in subjects at higher risk of cardiovascular disease (53, 55), increase in HDL-6 during GH-withdrawal also seems favorable. In adults, the effect of GH replacement on HDL-C levels is not fully clear at present (7). While a meta-analysis reported no significant effect on HDL-C levels (69), the study on HDL-subfractions found significant reductions in HDL-C especially within the large subfraction after six months of GH-replacement (43).

Although these findings are intriguing, it should be emphasized that these seemingly favorable shifts in lipoprotein subfractions have been observed after a short GH-withdrawal period. Prolonged GH-deficiency has a well-documented harmful effect on the lipid profile (1).

Previous studies consistently reported higher TC and LDL-C levels in AGHD patients, while HDL-C levels were frequently found unaltered (7). Similarly, while the favorable effect of GHRT on TC and LDL-C is well-documented, there is much less consistent evidence for the effect on HDL-C (7, 69). A key issue with majority of the previous studies is that they tended to focus solely on a quantitative analysis rather than a qualitative evaluation of the HDL particles. This has led to a conclusion that GH has no impact on HDL metabolism. In our study, we detected significant alterations in lipoprotein subfractions especially in the HDL subfractions of AGHD patients and found strong associations between IGF-1 and HDL subfractions, indicating a considerable role of GH/IGF-1 axis in HDL metabolism. Due to their role in reverse cholesterol transport as well as their anti-thrombotic, anti-inflammatory and antioxidant properties, HDLs are considered fundamental players in atheroprotection (9, 17). Since different HDL subfractions suggested to have different contribution to these HDL-related anti-atherogenic activities (16, 17), it is tempting to speculate that the observed alterations in HDL subfractions might indicate impaired HDL functionality in AGHD patients.

The main strengths of our study are the combined cross-sectional and prospective design as well as the use of high-quality methodology, which have allowed us to perform an in-depth analysis of lipoprotein subfraction levels and explore their associations in AGHD. Moreover, to the best of our knowledge, this is the first study to explore apoL1, apoM, and S1P levels in GH-deficient adults. We are aware that our work has some important limitations that must be addressed. One limitation is the small sample size of GHU and GHS patients, therefore our study should be considered a pilot and requires confirmation in future research. Another notable limitation is the higher BMI, waist circumference and insulin levels in our GHU patients, which can contribute to the alterations of lipoporotein subfractions. However, these are well-known characteristics of the so-called “GHD-syndrome”, making it very difficult to enroll unsubstituted GHD patients who are free from these metabolic complications. Heterogeneity in terms of etiology and the presence of other pituitary hormone deficiencies might also affect lipoprotein subfractions. To avoid the confounding effects of other replacement therapies, patients were included only if the concomitant pituitary deficiencies were adequately substituted. The inclusion of childhood and adult-onset GHD patients, wide variety of GHRT duration, presence of T2DM and the use of lipid-lowering medications may also confound the analysis and affect the generalizability of our findings. Measuring apoM-bound versus albumin-bound S1P could provide a clearer picture of the bioactive function of S1P. However, these measurements are time-consuming, costly and are not easily accessible in every day clinical practice. Additionally, longer GH-withdrawal might reveal more pronounced lipoprotein modifications, but may also lead to clinical deterioration, which we were trying to avoid. Aside from these limitations, we do believe that our findings provide a novel insight into the regulatory role of GH/IGF-1 axis in lipid metabolism.

To sum up, despite no changes in standard lipid parameters, we detected considerable alterations of lipoprotein subfractions in GH-deficient adults indicating that lipoprotein subfraction analysis may allow for a more accurate assessment of cardiovascular risk in AGHD. Compared to healthy individuals, distribution of HDL subfractions were particularly impaired in GH-unreplaced AGHD patients. Based on the strong associations found between HDL subfractions and log_10_IGF-1, physiologic IGF-1 levels might be required for normal HDL structure and functionality. Short-term GH-withdrawal resulted only subtle changes in lipoprotein subfractions, which seem to be favorable and possibly linked to improved insulin sensitivity.

In conclusion, our findings suggest that besides visceral obesity, insulin resistance and other well-documented risk factors, unfavorable distribution of lipid subfractions and the functional impairment of HDL particles may contribute to the increased cardiovascular risk observed in AGHD patients. These alterations underscore the importance of regular screening for cardiovascular complications and the implementation of effective lipid lowering therapies in the management of AGHD patients. Further large-scale, multicenter longitudinal studies are warranted to elucidate the clinical significance of the observed lipid metabolism abnormalities.

## Data Availability

The original contributions presented in the study are included in the article/**Supplementary Material**. Further inquiries can be directed to the corresponding author.
