# Supplementary material for: Alterations of lipoprotein subfractions in GH-deficient adults
Source: Front Endocrinol (Lausanne). 2025 Nov 5;16:1696426. doi: 10.3389/fendo.2025.1696426 (PMC12626791; doi:10.3389/fendo.2025.1696426)
Supplement: Supplementary file 1 [file Presentation1.pptx]

## Slide 1
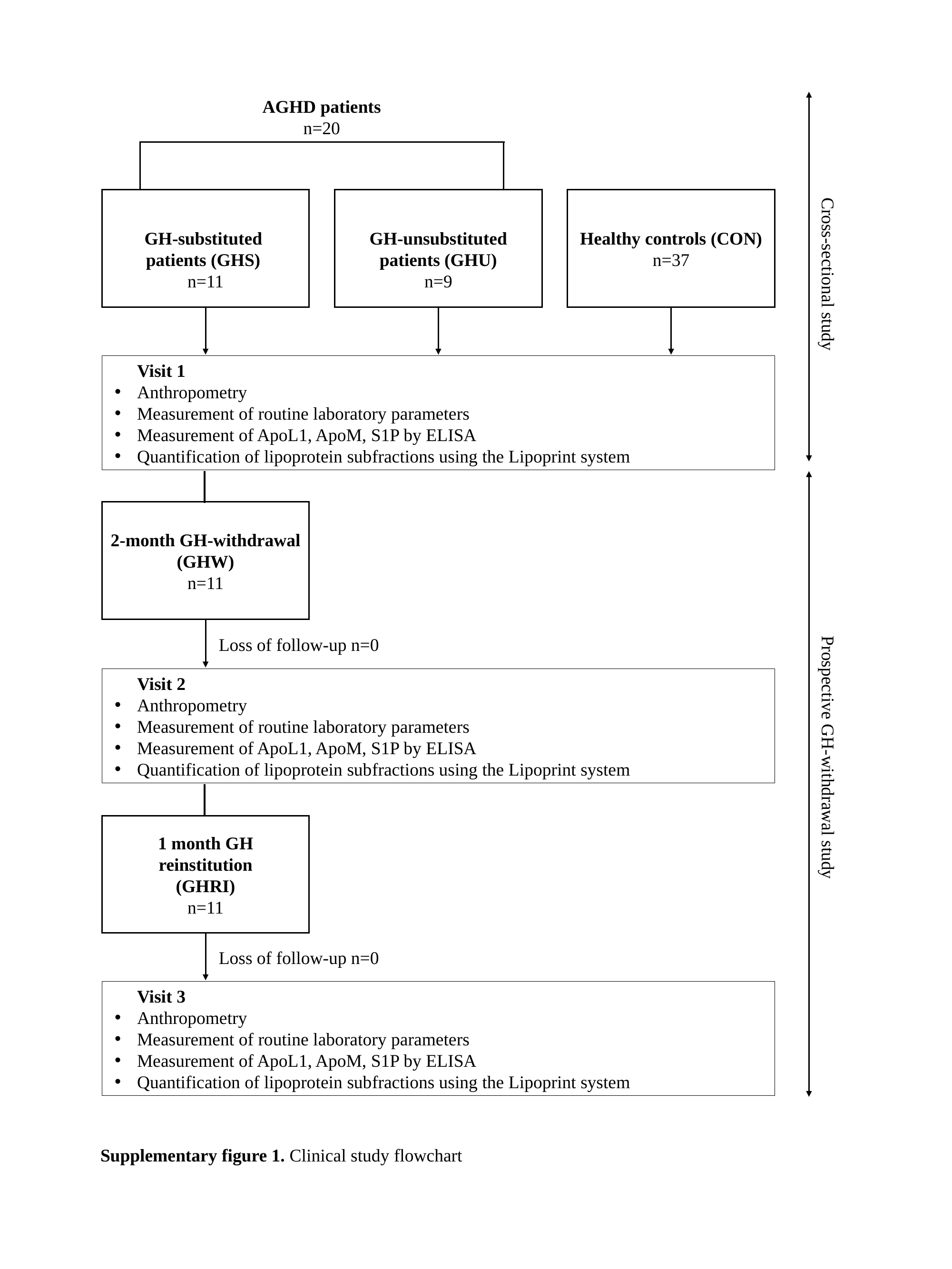

AGHD patients
n=20
GH-substituted
patients (GHS)
n=11
GH-unsubstituted patients (GHU)
n=9
Healthy controls (CON)
n=37
Cross-sectional study
Visit 1
Anthropometry
Measurement of routine laboratory parameters
Measurement of ApoL1, ApoM, S1P by ELISA
Quantification of lipoprotein subfractions using the Lipoprint system
2-month GH-withdrawal
(GHW)
n=11
Loss of follow-up n=0
Visit 2
Anthropometry
Measurement of routine laboratory parameters
Measurement of ApoL1, ApoM, S1P by ELISA
Quantification of lipoprotein subfractions using the Lipoprint system
Prospective GH-withdrawal study
1 month GH reinstitution
(GHRI)
n=11
Loss of follow-up n=0
Visit 3
Anthropometry
Measurement of routine laboratory parameters
Measurement of ApoL1, ApoM, S1P by ELISA
Quantification of lipoprotein subfractions using the Lipoprint system
Supplementary figure 1. Clinical study flowchart
