# Supplementary material for: Alterations of lipoprotein subfractions in GH-deficient adults
Source: Front Endocrinol (Lausanne). 2025 Nov 5;16:1696426. doi: 10.3389/fendo.2025.1696426 (PMC12626791; doi:10.3389/fendo.2025.1696426)
Supplement: Supplementary file 2 [file Table1.docx]

| **Supplementary Table 1.** Clinical characteristics of the enrolled AGHD patients. | | |
| --- | --- | --- |
|  | **GHU (n=9)** | **GHS (n=11)** |
| Male/female | 5/4 | 6/5 |
| Age, years mean (range) | 42.22 (21-61) | 43.18 (26-59) |
| COGHD/AOGHD | 3/9 | 5/11 |
| Other hormonal insufficiencies (n) |  |  |
| Thyroid | 9 | 11 |
| Corticosteroid | 8 | 8 |
| Gonadal | 8 | 7 |
| Antidiuretic | 3 | 5 |
| Other relevant medications (n) |  |  |
| statin | 2 | 0 |
| ezetimibe | 2 | 2 |
| fibrate | 2 | 0 |
| metformin | 1 | 2 |
| GLP-1-RA | 1 | 0 |
| SGLT2i | 1 | 0 |
| insulin | 0 | 1 |

Supplementary Material

AGHD, adult growth hormone deficiency; AOGHD, adult-onset growth hormone deficiency; COGHD, childhood-onset growth hormone deficiency; GHS, GH-substituted GH-deficient patients; GHU, GH-unsubstituted GH-deficient patients; GLP-1-RA, glucagon-like peptide 1 receptor agonist; SGLT2i; sodium-glucose cotransporter-2 inhibitor.

**
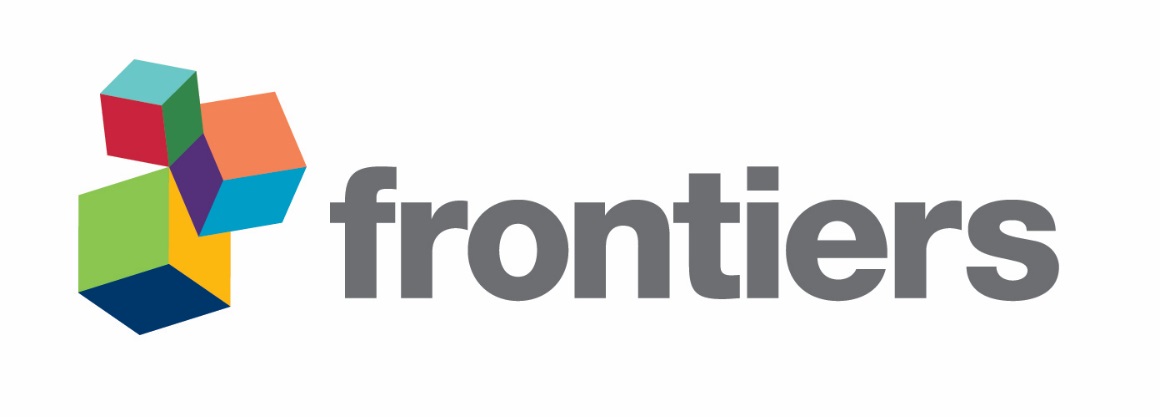
**
